# Feasibility and effects of horticultural activities on frailty, physical function, and quality of life among older adult residents in nursing homes: a quasi-experimental study

**DOI:** 10.3389/fpubh.2025.1562157

**Published:** 2025-07-14

**Authors:** Jingwen Wang, Xiaoyu Niu, Ruru Guo, Nana Liu, Weihong Zhang, Mingli Zhao, Jingjing Wang

**Affiliations:** ^1^School of Nursing and Health, Zhengzhou University, Zhengzhou, China; ^2^School of Medical Nursing, Shaanxi Energy Institute, Xianyang, China; ^3^Second Affiliated Hospital of Zhengzhou University, Zhengzhou, China; ^4^Institute of Medical and Pharmaceutical Research, Zhengzhou University, Zhengzhou, China; ^5^Department of Nursing, The Fifth People’s Hospital of Shanghai, Affiliated Fudan University, Shanghai, China

**Keywords:** frailty, older adult individuals, older adult care institutions, horticultural activities, physical function, quality of life

## Abstract

**Objectives:**

The aim was to evaluate the effectiveness of a horticultural activity intervention programme for improving frailty status, physical performance, and quality of life among frail older adult residents in nursing homes in Henan.

**Design:**

A quasi-experimental design was employed.

**Participants:**

Two nursing homes were selected through convenience sampling and were assigned to the intervention or control group via a random lottery. A total of 92 participants were recruited for this study, with 46 participants from the intervention institution and 46 participants from the control institution, on the basis of predefined inclusion and exclusion criteria.

**Methods:**

Both groups participated in regular nursing home activities. The intervention group additionally partook in a 6-month horticultural activity programme. Assessments of frailty, physical performance, and quality of life were assessed at baseline, 3 months, and 6 months post-intervention; validated instruments, including the Tilburg Frailty Indicator (TFI), the Chinese-Modified Physical Performance Test (CM-PPT), and the 12-Item Short Form Health Survey (SF-12), were used.

**Results:**

Compared with the control group, the intervention group demonstrated potential improvements in frailty (TFI scores), physical performance, and dimensions of quality of life, including general health, mental health, and physical health. These effects were supported by significant between-group, time, and interaction effects (*p* < 0.05).

**Conclusion:**

Horticultural activities may contribute to improvements in frailty, physical function, and quality of life in frail older adult residents in nursing homes, supporting the development of targeted interventions for this population. Future studies with larger sample sizes and subgroup analyses are recommended to compare effects across populations.

## 1 Introduction

Frailty is a prominent health issue among older adult residents in nursing homes and significantly affects physical function, leading to reductions in muscle strength, endurance, and balance, among other aspects (1, 2). In China, the prevalence of frailty among older adults has reached older adult 10.6% (3), and continues to rise due to increased longevity, lifestyle transitions, and chronic diseases burden (4). Rodriguez-Manas and Fried reported frailty prevalence ranging from 7 to 16.3% in industrialized countries, with higher rates among women and the oldest age group. Although China’s overall rate appears comparable, the absolute burden is considerable due to the large and rapidly aging population. Simultaneously, shifts in family structure and caregiving dynamics have led more older adults to enter nursing homes (5, 6), where frailty is particularly common-ranging from 44.3 to 59.2%-and often exacerbated by sedentary lifestyles and limited physical activity options (7, 8). Frailty in this setting is a key predictor of falls, hospitalization, disability, and mortality, significantly impairing quality of life (9, 10).

However, frailty is a dynamic and potentially reversible condition that can be improved by enhancing physical function in older adult individuals (11). Physical inactivity is a major risk factor for frailty, while moderate physical activity (PA) has been shown to alleviate frailty symptoms and improve physical resilience (10, 12, 13). Therefore, promoting regular PA is essential for maintaining the physical health of frail older adult residents in nursing homes and mitigating the progression of frailty.

Frailty is driven by physiological decline, including progressive loss of skeletal muscle mass, impaired neuromuscular coordination, and reduced cardiopulmonary capacity, which collectively reduce physical resilience and increase vulnerability to stressors (14, 15). These changes are especially pronounced in nursing home residents, who often face restricted access to structured physical activity and prolonged sedentary behavior. Sedentary behavior, defined as waking activity with energy expenditure below 1.5 METs in a sitting or reclining posture (16), further contributes to frailty progression. Therefore, interrupting sedentary routines is essential for delaying or reversing functional decline in this population.

The American Horticulture Therapy Association (AHTA) defines horticultural therapy (HT) as the use of structured gardening and plant-based activities to enhance physical, psychological, and social well-being (17). In China, where agrarian culture is deeply rooted, HAs provide a culturally appropriate form of light-to-moderate physical activity. Prior studies have shown that HAs can improve mobility, grip strength, cardiovascular function, and mental health among older adults (18–22). These multidimensional benefits make HAs a promising intervention for frailty, a condition with complex physical, psychological, and social components (23–25).

However, most existing studies on horticultural interventions have been conducted outside of the Chinese cultural context, where lifestyle habits, intergenerational dynamics, and views on aging differ considerably. Older adults in China often have strong ties to agricultural traditions and collective participation, making them potentially more receptive to horticultural activities. Despite this cultural compatibility, research on such interventions in Chinese nursing homes remains limited.

Therefore, this study aimed to evaluate the effectiveness of a culturally tailored HA intervention for frail older adult residents in Chinese nursing homes. We hypothesized that the intervention would improve frailty status, physical function, and quality of life by promoting light-to-moderate physical activity, enhancing emotional well-being, and increasing social engagement. The findings are expected to provide theoretical support for implementing HA-based interventions in institutional older adult care settings in China.

## 2 Methods

### 2.1 Study design

A two-arm quasi-experimental design with pre-test, mid-test, and post-test assessments was employed in this study. This study aimed to evaluate the effectiveness of an HA intervention programme in improving frailty status, physical performance, and quality of life among frail older adult residents in nursing homes in Henan. A convenience sampling method was used to select two nursing homes in Zhengzhou. A lottery was employed to determine which nursing home would receive the interventional protocol while the other nursing home would receive the “control protocol.” A total of 46 participants were selected from each of the two nursing homes by using the same inclusion and exclusion criteria. Older adult people in the control group maintained ordinary PA, and those in the experimental group participated in the HA intervention. These individuals received extra HA sessions twice per week, 60 min each time. The HAs included plant activities, handicraft activities, ornamental activities and derivative activities. The study was reported according to the Transparent Reporting of Evaluations with Nonrandomized Designs (TREND) checklist (26).

### 2.2 Study participants

This study was conducted in two nursing homes in China. The participants were included or excluded from the study according to the following criteria:

Inclusion criteria: (1) frail older adult individuals who had lived in the older adult care institution for more than 3 months (aged ≥60 years); (2) older adult individuals with a score ≥5 points on the Chinese version of the Tilburg Frailty Indicator (TFI); (3) older adult individuals who were conscious, had normal communication abilities, and could cooperate during the evaluation; (4) older adult individuals who volunteered to participate in the study; and (5) older adult individuals who could complete the walking test in this study.

Exclusion criteria: (1) frail older adult individuals who had severe vision or hearing disorders or difficultly communicating; (2) older adult individuals who had other critical diseases and could not cooperate with the intervention; (3) older adult individuals who had terminal diseases and an life expectancy <6 months; and (4) older adult individuals who were participating in other research simultaneously.

Sample size

$n=\frac{{\left(Z_{\alpha }+Z_{\beta }\right)}^{2}*2\sigma^{2}}{\delta^{2}}$

In this study, frailty level was taken as the primary outcome indicator, and the sample size was calculated according to the formula. The sample ratio between the intervention group and the control group was 1:1, *σ*^2^ was the overall variance, and the sample variance, *S*^2^, was used for estimation, with an *α* = 0.05 and *β* = 0.1 (27). This ratio was calculated using a *δ*^2^ = 1.1236 and *σ*^2^ = 2.91, and the above data were substituted into the formula to obtain *n* ≈ 44. Considering a 5% loss to follow-up rate, the sample sizes of the intervention group and the control group were 46 patients, for a total of 92 patients overall. All the subjects provided informed consent. This study was reviewed and approved by the Life Sciences Ethics Review Committee of Zhengzhou University (ZZUIRB2021-54).

### 2.3 Construction of the intervention plan

#### 2.3.1 Literature research

To inform the design of the HA intervention, we conducted a targeted literature review using content analysis. Literature searches were performed in both Chinese and English databases, including CNKI, Wanfang, PubMed, and Web of Science, covering publications from January 2010 to 2021. We used the following keywords in various combinations: “horticultural therapy,” “horticultural activities,” “frailty,” “older adult,” and “nursing home.” The inclusion criteria were: (1) studies involving structured horticultural or plant-based interventions; (2) studies targeting older adult or frail older adult populations; and (3) studies evaluating physical, psychological, or social outcomes. After screening titles and abstracts, removing duplicates and irrelevant studies, a total of 19 articles were retained for full-text review and synthesis. These studies were used to guide the selection of activity types, implementation frequency, duration, and thematic elements in our final intervention plan.

#### 2.3.2 Expert panel meeting

A total of 9 experts in relevant research fields-including geriatric nursing care, community healthcare nursing, and rehabilitation care-were invited to participate in the development of the first draft of the intervention plan. The panel included two geriatric nursing specialists, three community health nurses, two rehabilitation therapists, and two senior nursing home managers, all with over 10 years of experience in their respective fields. The expert panel meeting was conducted online. Based on the feedback collected during the meeting, the intervention programme was revised and refined to improve its content validity, feasibility, and applicability in institutional care settings (Supplementary Table S1).

### 2.4 Intervention

The intervention group participated in a structured HA program over 6 months, with two sessions per week, each lasting 60 min. Activities included planting, handicrafts, ornamental gardening, and related tasks, delivered in a group format. The intensity ranged from light to moderate, tailored to participants’ abilities. Each session followed a three-part structure: warm-up, main activity, and summary (28). A detailed description of the activity themes, intensity levels, and objectives is provided in Supplementary Table S2. The control group continued routine sedentary activities such as watching TV, playing mahjong, and walking. Assessments of frailty, physical function, and quality of life were conducted at baseline, 3 months, and 6 months. After the intervention period, the control group received a compensatory HA program.

### 2.5 Study measures

The Chinese version of the TFI, the Chinese Mini-Physical Performance Test (C-MPPT), and the 12-item Short Form Health Survey (SF-12) were used to evaluate the intervention protocol among the older adult residents at three time points (before the intervention, at the third month of the intervention, and at the sixth month of the intervention). The contents of the assessment were as follows:

#### 2.5.1 Basic characteristics

The basic characteristics included sex, age, education level, marital status, personal monthly income, number of chronic illnesses, number of children, and length of stay in the nursing home.

#### 2.5.2 Primary outcomes

Frailty was assessed as the primary outcome using the TFI, which was developed by Dutch scholar Gobbens et al. (29). The Chinese version of the TFI (30) consists of 15 items that encompass three dimensions: physical frailty (8 points), psychological frailty (4 points), and social frailty (3 points). The total score of the scale ranges from 0 to 15 points, with higher scores indicating greater frailty. A score of 5 points or above indicates frailty. The TFI has demonstrated good reliability and validity in its application among older adult individuals residing in nursing homes (31).

#### 2.5.3 Secondary outcomes

The C-MPPT (32) on the basis of the Physical Performance Test (33). The C-MPPT comprises four items: balance while standing, the chair stand test, the 6-metre walk test, and repeated chair stands. Each item is scored from 0 to 4, yielding a total score ranging from 0 to 16 points. A higher score indicates better physical function.

The SF-12 is a shortened version of the widely used and concise Short Form-36 (SF-36) quality of life instrument developed by the Boston Health Institute in the United States (34). The SF-12 was designed to evaluate both physical and mental health status. The scale consists of 12 items that assess eight dimensions of health-related quality of life, including general health, physical functioning, role-physical, bodily pain, vitality, social functioning, role-emotional, and mental health. Scores for each domain are standardized to range from 0 to 100, with higher scores indicating better quality of life. The SF-12 has shown acceptable psychometric properties among Chinese older adult populations, with a Cronbach’s alpha coefficient of 0.775 and structural validity confirmed by factor analysis (KMO = 0.824; cumulative variance contribution rate = 58.1%) in a sample of 451 older adults (35).

Data were collected at three time points: baseline (T0), at the third month (T1), and at the end of the 6-month intervention (T2). The data collectors included the researchers, 2 nurses in nursing homes and 2 care workers in nursing institutions. Before data collection, the researchers trained the data collection personnel, including the purpose of questionnaire completion, the specific content of the questionnaire, the evaluation methods of subjective and objective indicators and matters needing attention. All questionnaires were collected on the spot and checked to reduce the generation of invalid questionnaires.

### 2.6 Statistical analysis

Measurement data with a normal distribution are presented as the means and standard deviations, whereas measurement data without a normal distribution are presented as medians and quartiles. The categorical data are presented as frequencies and percentages, and intervention effects were evaluated. Generalized estimating equations (GEEs) were employed to analyse the between-group effects, time effects, and interaction effects between the two groups. Data entry was performed by two individuals using Epidata 3.1 using a two-sided test with an *α* = 0.05 indicating significance, and statistical analysis was conducted using SPSS 22.0 software.

## 3 Results

### 3.1 Sample characteristics

A total of 92 study participants were included in this study (46 in the intervention group and 46 in the control group). One participant in the intervention group and 2 participants in the control group were lost to follow-up. Ultimately, a total of 89 participants participated in this study (45 in the intervention group and 44 in the control group), and the overall loss to follow-up rate was 3.26% (<20%). There was no significant difference in the general data between the two groups (*p* > 0.05) (Table 1).

**Table 1:** Comparison of baseline characteristics between the intervention and control group.

Variables	Intervention group (*n* = 45) *n* (%)	Control group (*n* = 44) *n* (%)	*p*
Sex			
Female	35 (77.8)	32 (72.7)	
Age (years, x̄ ± s)	84.09 ± 7.166	83.34 ± 6.762	0.891
Education level			
Under primary school	15 (33.3)	19 (43.2)	*p* = 0.270
Junior middle school	5 (11.1)	6 (13.6)	
Senior high school/polytechnic school	11 (24.4)	8 (18.2)	
Junior college	6 (13.3)	6 (13.7)	
Bachelor degree or above	8 (17.8)	5 (11.4)	
Marital status			
Married	12 (26.1)	14 (31.9)	0.730
Monthly income (CNY)			
≤1,000	8 (17.8)	7 (15.9)	0.103
1,001–2000	0 (0)	3 (6.8)	
2,001–3,000	5 (11.1)	12 (27.3)	
>3,000	32 (71.1)	22 (50.0)	
Number of children			
0	1 (2.2)	0 (0)	0.789
1	4 (8.9)	2 (4.5)	
2	8 (17.8)	9 (20.5)	
≥3	32 (71.1)	33 (75.0)	
Duration of nursing home stay (years)			
0~	10 (22.2)	11 (24.4)	0.136
1~	5 (11.1)	7 (15.9)	
2~	8 (17.8)	12 (27.3)	
3~	4 (8.9)	4 (9.0)	
4~	7 (15.6)	6 (13.6)	
5~	11 (24.4)	4 (9.0)	
Number of chronic diseases			
0	6 (13.3)	2 (4.5)	0.495
1	15 (33.3)	15 (33.3)	
2	13 (28.9)	16 (36.4)	
≥3	11 (24.4)	11 (25.0)	

### 3.2 Comparison of frailty between the two groups

There was no statistically significant difference in the frailty scale scores before the intervention between the two groups (*p* > 0.05), indicating comparability. There were significant differences in the intergroup effect, time effect and interaction effect of the total score and the psychological frailty score (*p* < 0.05). There was a significant difference in the interaction effect of the physical frailty score (*p* < 0.05), but there was no significant difference in the time effect or intergroup effect (*p* > 0.05). There were significant differences between the groups and interaction effects in terms of the social frailty score (*p* < 0.05), but there were no significant differences in the time effect (*p* > 0.05) (Table 2).

**Table 2:** Generalized estimation equation analysis of TFI scores of two groups of subjects (*n* = 89).

Variables	Mean (SD)		Inter-group effect	Time effect	Group*time effect
	IG	CG	*p*	*p*	*p*
Total score			<0.001	<0.001	<0.001
T0	10.04 (2.54)	9.98 (1.99)			
T1	8.84 (2.04)	10.48 (1.84)			
T2	7.42 (2.20)	11.25 (2.04)			
Physical frailty			0.128	0.060	<0.001
T0	5.64 (1.76)	5.55 (1.47)			
T1	5.27 (1.51)	5.66 (1.31)			
T2	4.82 (1.45)	5.86 (1.34)			
Psychological frailty			<0.001	0.005	<0.001
T0	2.56 (1.04)	2.619 (0.99)			
T1	1.98 (0.78)	2.82 (0.82)			
T2	1.339 (0.77)	3.14 (0.77)			
Social frailty			<0.001	0.629	<0.001
T0	1.84 (0.60)	1.829 (0.58)			
T1	1.60 (0.54)	2.00 (0.61)			
T2	1.27 (0.50)	2.25 (0.62)			

T0, baseline; T1, at the third month of the intervention; T2, at the sixth month of the intervention; IG, Intervention group; CG, control group.

In summary, the intervention is effective because the frailty level of the intervention group shows a decreasing trend over time, while that of the control group increases. Over time, the differences between the two groups are particularly evident in the interaction effect, and these parameters indeed assess changes through measurements at multiple time points.

### 3.3 Comparison of physical function between the two groups

There was no statistically significant difference in the total physical function score or the scores of each dimension between the two groups before the intervention (*p* > 0.05), indicating comparability. A GEE analysis revealed statistically significant differences in the intergroup effect, time effect and interaction effect of the total physical function score, standing-static balance score and 6-M walking score (*p* < 0.05). The interaction effect of the sit down–stand-up score was statistically significant (*p* < 0.05), but there was no significant difference between the time effect and the intergroup effect (*p* > 0.05). The intergroup effect and interaction effect of the Squat score were statistically significant (*p* < 0.05), but the difference in the time effect was not statistically significant (*p* > 0.05) (Table 3).

**Table 3:** Generalized estimation equation analysis of body function scores of two groups of subjects (*n* = 89).

Variables	Mean (SD)		Inter-group effect	Time effect	Group*time effect
	IG	CG	*p*	*p*	*p*
Physical function total			<0.001	0.001	<0.001
T0	6.49 (1.67)	6.25 (2.21)			
T1	7.47 (2.02)	5.89 (1.57)			
T2	8.89 (2.29)	5.48 (1.82)			
Standing-static balance			0.001	<0.001	<0.001
T0	2.04 (0.80)	1.98 (0.70)			
T1	2.44 (0.89)	2.00 (0.72)			
T2	2.71 (0.87)	1.84 (0.78)			
Sit down-stand up			0.052	0.551	0.024
T0	1.44 (1.06)	1.32 (0.88)			
T1	1.60 (1.05)	1.25 (0.89)			
T2	1.71 (0.87)	1.20 (0.90)			
The 6 m walking			<0.001	0.001	<0.001
T0	1.62 (0.91)	1.64 (0.84)			
T1	2.18 (1.09)	1.59 (0.79)			
T2	2.69 (0.85)	1.39 (0.62)			
Squat			0.025	0.193	<0.001
T0	1.38 (1.01)	1.32 (0.91)			
T1	1.44 (1.06)	1.14 (0.67)			
T2	1.78 (0.97)	1.05 (0.61)			

T0, baseline; T1, at the third month of the intervention; T2, at the sixth month of the intervention; IG, Intervention group; CG, control group.

In summary, the intervention is effective: IG shows improved physical function over time, while CG does not. Interaction effects highlight divergent group-time trends, and multi-time-point measurements confirm parameter-level change assessment.

### 3.4 Comparison of quality of life between the two groups

There was no statistically significant difference in the scores on all quality of life dimensions between the two groups before the intervention (*p* > 0.05), indicating comparability. The intergroup effect, time effect and interaction effect of the general health, physical functioning, mental health, mental component summary score and physical component summary scores of the two groups were statistically significant (*p* < 0.05). The time effect and interaction effect of role–emotional score were statistically significant (*p* < 0.05), but there was no significant difference between the groups (*p* > 0.05). The intergroup effect and interaction effect of the vitality and social functioning scores were statistically significant (*p* < 0.05), but the time effect was not statistically significant (*p* > 0.05) (Table 4).

**Table 4:** Analysis of generalized estimation equation for QOL scores of the two groups of subjects (*n* = 89).

Variables	Mean (SD)		Inter-group effect	Time effect	Group*time effect
	IG	CG	*p*	*p*	*p*
General health (GH)			0.026	0.039	<0.001
T0	27.78 (15.28)	27.84 (13.44)			
T1	31.67 (20.23)	26.70 (13.64)			
T2	40.56 (24.59)	23.86 (15.21)			
Physical functioning (PF)			0.005	0.026	<0.001
T0	36.11 (28.98)	35.80 (20.46)			
T1	48.33 (27.39)	32.39 (23.24)			
T2	53.33 (26.44)	29.55 (24.87)			
Role-physical (RP)			0.514	0.949	0.645
T0	17.78 (33.97)	17.05 (26.29)			
T1	18.89 (35.82)	14.77 (25.47)			
T2	20.00 (32.68)	13.64 (22.53)			
Role-emotional (RE)					
T0	50.00 (42.64)	48.86 (42.44)	0.097	0.005	<0.001
T1	57.78 (43.90)	47.73 (43.07)			
T2	73.33 (31.26)	44.32 (43.42)			
Bodily pain (BP)					
T0	60.56 (29.91)	60.23 (28.69)	0.669	0.749	0.931
T1	61.67 (24.77)	59.66 (29.64)			
T2	61.11 (27.98)	56.82 (26.06)			
Mental health (MH)			<0.001	<0.001	<0.001
T0	45.56 (16.31)	51.36 (14.40)			
T1	77.56 (17.60)	49.09 (11.17)			
T2	82.67 (13.72)	48.18 (8.70)			
Vitality (VT)			0.006	0.071	<0.001
T0	49.78 (20.28)	50.91 (25.68)			
T1	52.89 (24.92)	46.82 (19.74)			
T2	66.67 (23.36)	42.73 (16.48)			
Social functioning (SF)			<0.001	0.450	<0.001
T0	60.89 (22.95)	59.55 (23.82)			
T1	68.00 (22.72)	54.09 (22.65)			
T2	76.89 (14.11)	49.09 (20.89)			
Mental component summary (MCS)			<0.001	<0.001	<0.001
T0	50.04 (15.26)	52.893 (16.44)			
T1	67.980 (17.52)	49.587 (12.96)			
T2	76.869 (14.37)	46.798 (10.40)			
Physical component summary (PCS)			0.009	0.025	<0.001
T0	38.730 (19.94)	39.123 (16.77)			
T1	44.127 (23.01)	37.013 (13.54)			
T2	53.175 (21.51)	32.468 (13.99)			

T0, baseline; T1, at the third month of the intervention; T2, at the sixth month of the intervention; IG, Intervention group; CG, control group.

In summary, the intervention is effective: IG shows improved quality of life over time, while CG does not. Interaction effects highlight divergent group-time trends, and multi-time-point measurements confirm parameter-level change assessment.

These results suggest that the intervention group experienced improvements in several dimensions of quality of life over the intervention period, including general health, physical functioning, mental health, and both component summary scores. In contrast, the control group did not exhibit such improvements. The significant time-by-group interaction effects confirm that the observed changes were primarily attributable to the intervention rather than natural fluctuation or external factors. This means the positive impact of the horticultural activity program on the physical and psychological well-being of frail older adult participants.

## 4 Discussion

This study investigated the effects of the HAs employed on changes in terms of frailty: frailty status, physical performance, and quality of life. The results suggest targeted improvements in frailty status (particularly psychological and social dimensions), as well as specific aspects of physical performance-most notably static balance and walking ability-following the implementation of the intervention activities. Specifically, the total TFI score and domain-specific frailty scores showed notable reductions in the intervention group. HAs were associated with beneficial changes in somatic and social frailty scores. Importantly, HAs were found to be more effective than casual activities in terms of the total physical functioning score, standing still-balance score and 6-M walking score. Furthermore, participating in HAs led to noticeable improvements in specific aspects of quality of life-particularly mental health and psychological well-being-rather than improvements across all dimensions.

HAs are beneficial for enhancing the psychological and social well-being of older adult individuals residing in nursing homes, with observed improvements primarily in mental health and social interaction domains. The rationale behind this assertion is outlined as follows:

1. Engaging in activities such as planting and caring for plants reduces sedentary behavior and increases overall PA levels, which is advantageous for addressing the physical frailty often experienced by this demographic (36).
2. HAs may contribute to improved mental well-being, as reflected by reduced psychological frailty and improved mental health scores in our results. Such activities may help mitigate feelings of loneliness and enhance overall enjoyment, thereby contributing positively to mental health among older adults (37).
3. The intervention model employed in this study was based on group participation, which fostered collaboration among the participants and encouraged involvement from family members. This approach facilitates greater social support for older adult individuals, effectively reducing their sense of social isolation. Consequently, it enhances their engagement in social activities and improves their overall state of social well-being (38).

The horticultural intervention programme was shown to improve the quality of life of older adult individuals residing in nursing homes. The underlying reasons are articulated as follows:

1. From a physiological perspective, engaging in HAs may improve the physical capabilities of frail older adult individuals, as reflected in enhanced static balance and 6-meter walking performance observed in our study. These improvements suggest that such activities may promote greater physical activity and engagement in body movement. While previous studies have proposed potential benefits such as improved blood circulation and cardiopulmonary function, our study did not directly assess these physiological parameters. Further research is needed to validate these mechanisms using objective biological or clinical indicators. Additionally, HAs involve multiple senses-sight, hearing, taste, touch, and smell-which may collectively contribute to physical and sensory stimulation in older adult participants (39).
2. From a psychological standpoint, gardening activities can improve cognitive function among older adult people by stimulating their senses and enhancing memory retention while improving their concentration and critical thinking skills (40). These activities provide an effective means for frail seniors to mitigate negative emotions by redirecting their focus towards new experiences associated with gardening; this diversion allows them to temporarily set aside adverse feelings. Additionally, horticultural engagement promotes positive emotional states among vulnerable older adults. The inherent benefits and natural beauty exhibited by plants contribute to reduced sympathetic nerve activity while increasing parasympathetic nerve activity; this shift results in improved heart rate regulation and overall emotional well-being. Moreover, witnessing plant growth firsthand-alongside harvesting produce or creating handicrafts-and receiving affirmation from peers fosters feelings of satisfaction, confidence, achievement, and self-efficacy among individuals who engage with horticulture (41).
3. From a social dimension perspective, the group intervention in this study aimed to encourage frail older adult individuals to engage in communication, cooperation, assistance, and sharing with their peers. Additionally, the intervention promoted the involvement of family members to enhance common topics and concerns between older adult individuals and their families. This research also supports the participation of staff from elder care institutions to foster connections across multiple dimensions, thereby enabling older adults to access greater social support. Such initiatives are beneficial in transforming the social engagement of older adult people and enhancing their participation in community activities (42).

## 5 Limitations

The generalizability of this study’s findings is limited, as participants were not representative of the broader frail older adult population in nursing homes. First, due to the COVID-19 pandemic and associated home-isolation policies in China, participant enrolment was restricted, potentially affecting sample diversity. Although facilitators ensured structured implementation, the intervention was designed to maintain light-to-moderate PA levels, and climate variability in early spring led to more indoor sessions. Some in-place exercises were included to compensate, but these may have provided limited somatic stimulus for broader frailty improvement, particularly in lower-limb function.

Additionally, the intervention emphasized upper-body engagement through planting, watering, and handcrafting, which may have improved adherence and fine motor function, but offered less stimulus for activities requiring balance or sit-to-stand ability. Another limitation is the reliance on self-reported instruments for key outcomes, such as the TFI, which may introduce subjective bias. While the CM-PPT provided some objective assessments (walking speed, lower-limb strength), we did not employ direct behavioral tracking tools (pedometers or actigraphy) or biomarker-based measures (handgrip strength, gait speed) to quantify PA levels or frailty components.

However, horticultural activity interventions appear cost-effective and feasible for institutionalized older adult populations, provided sufficient space and staffing support. Future studies should incorporate direct tracking tools, standardized frailty phenotypes, or biological indicators to improve methodological rigor. Multicentre trials with larger sample sizes, expanded outdoor modules, and subgroup analyses will also help address residual confounding and validate findings across settings.

## 6 Conclusion

The HA intervention programme designed in this study is culturally appropriate, scientifically grounded, and practically feasible for older adult populations in China. The implementation of HAs may contribute to improvements in frailty status, physical function, and quality of life among older adult populations in nursing homes and can provide a reference for older adult care institutions to develop feasible intervention programmes that may help address the debilitation of the older adult population. However, this study also has shortcomings. Owing to the limitations of manpower and material resources, the sample size of this study was small, and the selection of research objects was limited, which warrants cautious interpretation of the study findings and highlights the need for further confirmatory research. In future horticultural intervention studies, the sample size can be increased, and subgroup analysis can be conducted for confounding factors to compare whether the intervention has the same effect on these factors separately.

## Data Availability

The raw data supporting the conclusions of this article will be made available by the authors, without undue reservation.
