# Supplementary material for: Feasibility and effects of horticultural activities on frailty, physical function, and quality of life among older adult residents in nursing homes: a quasi-experimental study
Source: Front Public Health. 2025 Jul 14;13:1562157. doi: 10.3389/fpubh.2025.1562157 (PMC12301357; doi:10.3389/fpubh.2025.1562157)
Supplement: Supplementary file 1 [file Table_1.docx]

**Supplementary Table S1** Revision content of the trial test intervention plan

| **Open question** | **Modified content** |
| --- | --- |
| The survival rate of strawberry and tomato seedlings raised with seeds is low | When implementing the intervention program, it is recommended to directly buy the seedlings that have been cultivated, which helps to improve the survival rate. |
| Poor ventilation conditions affect the survival rate and growth rate. | It is recommended to choose a site with good ventilation conditions. |
| The lack of activity space affects the enthusiasm and implementation progress of the participants. | It is recommended to choose a space with sufficient activity space. |
| The completion degree of some activities is not high, and the implementation personnel fail to fully implement the activity content according to the activity plan. | Implementation personnel should be trained before the activity. Each activity should be supervised by researchers to supervise the activity and urge the executor to carry out the activity plan. |
| Llettuce has in lettuce. | Conduct regular deworming activities and plant lettuce on high places. |
| In the theme activities of "Sow seedlings" and "Strong lettuce", three kinds of plants were bred and planted at the same time, and the workload is heavy. In addition, it is not conducive to the management of plants. | Breeding and planting in batches. |
