# Supplementary material for: Feasibility and effects of horticultural activities on frailty, physical function, and quality of life among older adult residents in nursing homes: a quasi-experimental study
Source: Front Public Health. 2025 Jul 14;13:1562157. doi: 10.3389/fpubh.2025.1562157 (PMC12301357; doi:10.3389/fpubh.2025.1562157)
Supplement: Supplementary file 2 [file Table_2.docx]

**Supplementary Table S2 Horticultural activity intervention program for frail elderly residents in nursing homes**

| Times per week | Theme | Content | Intensity | Object |
| --- | --- | --- | --- | --- |
| 1 | Recall the years of cultivation | Activity 1：①Mobilization meeting;②Describe the experience of participating in HAs | Low intensity  (Level 3-4) | B+C |
|  |  | Activity 2：①Safety education;②Grouping;③Play a game: Guess what's in the botanical garden? | Medium intensity  (Level 5-6) | A+C |
| 2 | Open up new horizons | Activity 1: Divide the area; Sorting out the soil | Medium intensity  (Level 5-6) | A+C |
|  |  | Activity 2: Prepare pot soil | Medium intensity  (Level 5-6) | A+C |
| 3 | Sow seedlings | Activity 1: Planting strawberry seedlings; | Medium intensity  (Level 5-6) | A+C |
|  |  | Activity 2: Planting tomato seedlings | Medium intensity  (Level 5-6) | A+C |
| 4 | Beautiful lavender | Activity 1: Planting lavender seedlings | Medium intensity  (Level 5-6) | A+C |
|  |  | Activity 2: ① Pruning lavender seedlings; ② sunflower seedling cultivation | Medium intensity  (Level 5-6) | A+C |
| 5 | Sunflower growth | Activity 1: Prepare pot soil | Medium intensity  (Level 5-6) | A+C |
|  |  | Activity 2: Planting sunflower seedlings; | Medium intensity  (Level 5-6) | A+C |
| 6 | The scent of "wormwood" | Activity 1: Prepare pot soil, trim and treat wormwood seedlings | Medium intensity  (Level 5-6) | A+C |
|  |  | Activity 2: Planting wormwood seedlings; | Medium intensity  (Level 5-6) | A+C |
| 7 | Qingming Festival | Activity 1: Making kites | Medium intensity  (Level 5-6) | A+B+C |
|  |  | Activity 2: Fly kites; | Medium intensity  (Level 5-6) | A+B+C |
| 8 | Carnations for Mother's Day | Activity 1: Making paper carnations | Low intensity  (Level 3-4) | A+B+C |
|  |  | Activity 2: ① Make paper carnations; ②Share | Medium intensity  (Level 5-6) | A+B+C |
| 9 | Strong lettuce | Activity 1: Prepare pot soil | Medium intensity  (Level 5-6) | A+C |
|  |  | Activity 2: Planting lettuce | Medium intensity  (Level 5-6) | A+C |
| 10 | Care plants | Activity 1: ① weeding, loosening, watering, fertilizing; ② Prepare pot soil | Medium intensity  (Level 5-6) | A+C |
|  |  | Activity 2: Move flowerpots, pruning, scaffolding, fertilization, placing flowerpots | Medium intensity  (Level 5-6) | A+C |
| 11 | Dragon Boat Festival | Activity 1: ① Make wormwood sachet; ② Share | Low intensity  (Level 3-4) | A+B+C |
|  |  | Activity 2: ① Make rice dumplings;② Share | Medium intensity  (Level 5-6) | A+B+C |
| 12 | Care plants | Activity 1: ① weeding, loosening, watering, fertilizing; ② Prepare pot soil | Medium intensity  (Level 5-6) | A+C |
|  |  | Activity 2: Move flowerpots, pruning, scaffolding, fertilization, placing flowerpots | Medium intensity  (Level 5-6) | A+C |
| 13 | Green food | Activity 1: ① Harvest lettuce; ② weeding, loosening, watering; | Medium intensity  (Level 5-6) | A+B+C |
|  |  | Activity 2: ① Make vegetable salad; ② Share the idea of placing the plate; | Low intensity  (Level 3-4) | A+B+C |
| 14 | Care plants | Activity 1: ① weeding, loosening, watering, fertilizing; ② Prepare pot soil | Medium intensity  (Level 5-6) | A+C |
|  |  | Activity 2: Move flowerpots, pruning, scaffolding, fertilization, placing flowerpots | Medium intensity  (Level 5-6) | A+C |
| 15 | Self-sufficiency | Activity 1: ① Carry the soil, prepare the basin soil, and place the flower pot | Medium intensity  (Level 5-6) | A+C |
|  |  | Activity 2: ① Make fruit platter; ② Share ideas and ideas | Low intensity  (Level 3-4) | A+B+C |
| 16 | Vitality | Activity 1: Prepare pot soil | Medium intensity  (Level 5-6) | A+C |
|  |  | Activity 2: Planting carrot seedlings | Medium intensity  (Level 5-6) | A+C |
| 17 | The sun flowers bloom | Activity 1: Prepare the materials for the sunflower bouquet | Medium intensity  (Level 5-6) | A+C |
|  |  | Activity 2: ① Harvest sunflowers; ② Make a bouquet of sunflowers | Medium intensity  (Level 5-6) | A+B+C |
| 18 | Care plants | Activity 1: ① weeding, loosening, watering, fertilizing; ② Prepare pot soil | Medium intensity  (Level 5-6) | A+C |
|  |  | Activity 2: Move flowerpots, pruning, scaffolding, fertilization, placing flowerpots | Medium intensity  (Level 5-6) | A+C |
| 19 | Harvest season | Activity 1: Prepare the ingredients | Medium intensity  (Level 5-6) | A+C |
|  |  | Activity 2: ① Make yogurt fruit ; ② Share | Medium intensity  (Level 5-6) | A+B+C |
| 20 | Full of vitality | Activity 1: Transplanting and pruning longevity flowers | Medium intensity  (Level 5-6) | A+C |
|  |  | Activity 2: Drawing longevity flowers | Low intensity  (Level 3-4) | A+B+C |
| 21 | Care plants | Activity 1: ① weeding, loosening, watering, fertilizing; ② Prepare pot soil | Medium intensity  (Level 5-6) | A+C |
|  |  | Activity 2: Move flowerpots, pruning, scaffolding, fertilization, placing flowerpots | Medium intensity  (Level 5-6) | A+C |
| 22 | Happy atmosphere | Activity 1: Pick carrots | Low intensity  (Level 3-4) | A+B+C |
|  |  | Activity 2: ① Make carrot meal; Play games; ② Share | Medium intensity  (Level 5-6) | A+B+C |
| 23 | Sweet dream sachets | Activity 1: Prepare materials for making lavender sachets | Low intensity  (Level 3-4) | A+C |
|  |  | Activity 2: ① Make lavender sachets; ② Share | Medium intensity  (Level 5-6) | A+B+C |
| 24 | Summary ceremony | Activity 1: ① Maintenance of longevity flower; ② Give each other longevity flowers | Medium intensity  (Level 5-6) | A+C |
|  |  | Activity 2: ① Handmade longevity flowers; ② Display and share; ③ Group photo | Medium intensity  (Level 5-6) | A+B+C |

**Note:**①intervention aims: A= increase the amount of PA; B= enhanced self-efficacy; C= Increase social participation.

②Activity intensity can be expressed as self-perceived exercise intensity (ratings of perceived exertion, RPE).
